# 

**Published:** 1949-01

**Authors:** 


### The Bristol Medico-Chirurgical Journal

A JOURNAL OF THE MEDICAL SCIENCES FOR THE

### West of England and South Wales

PUBLISHED UNDER THE AUSPICES OF

*THE BRISTOL MEDICO-CHIRURGICAL SOCIETY*

EDITOR

E. WATSON-WILLIAMS, *M.C.*, M.D., Ch.M., F.R.C.S.

WITH WHOM ARE ASSOCIATED

G. E. F. SUTTON, *M.C.*, M.D., B.S., F.R.C.P.

W. MELVILLE CAPPER, M.B., B.S., F.R.C.S.

J. M. NAISH, M.D., M.R.C.P.

N. S. CRAIG, *M.C.*, M.D., *Assistant Editor*

"Scire est nescire, nisi id me

Scire alius sciret"

1949 *VOL. LXVI*

BRISTOL: J. W. ARROWSMITH LTD.

WI
BR323

### 1949 Contents of Vol. LXVI.

| | Page |
|---|---|
| Obstetrics and Gynæcology: Old and New. By H. J. Drew Smythe, M.C., T.D. | 1 |
| The Thirty-seventh Long Fox Memorial Lecture: Some Aspects of Hospital Economy. By R. Milnes Walker, M.S. | 7 |
| The Quadruplets. I.—Delivery: by H. L. Shepherd, Ch.M., M.B., F.R.C.O.G. and Mabel F. Potter, M.D., F.R.C.O.G. II.—Management: by Beryl D. Corner, M.D., M.R.C.P. III.—Notes: by E. G. Watson-Williams, B.A. | 14 |
| Early Life. By A. V. Neale, M.D., F.R.C.P. | 31 |
| Arteriography of the Lower Limbs. By H. D. Moore, F.R.C.S. | 37 |
| The Surgery of Vascular Disease. By R. Milnes Walker, M.S., F.R.C.S. | 42 |
| The Second Carey Coombs Memorial Lecture. The Radiology of Rheumatic Heart Disease. By Sir John Parkinson, M.D., F.R.C.P. | 46 |
| Psychogenic Rheumatism: Its Differentiation from True Rheumatic Disease. By Philip S. Hench, M.D. | 48 |
| The Problem of Cut Flexor Tendons in Fingers. By A. L. Eyre-Brook | 63 |
| The Dramatic in Gynæcology and Obstetrics. By V. B. Green-Armytage | 69 |
| Cholecystitis due to Liver Fluke. By G. P. O'Donnell | 74 |
| Some Diseases of the Newborn Baby. By A. A. Moncrieff | 77 |
| Unusual Children. I.—Congenital Syphilis with Gangrene of the Extremities. II.—Dwarfism. III.—Progeria: by A. V. Neale, M.D., F.R.C.P. IV.—Cretinism. V.—Mongolism: by Beryl D. Corner, M.D., M.R.C.P. VI.—Gargoylism: by John Lowe, M.D., F.R.C.P. | 89 |
| The Prophylactic Uses of Chemotherapy. By J. A. Boycott, D.M. | 95 |
| Thymectomy for Myasthenia Gravis. By Geoffrey Keynes, M.D., F.R.C.S. | 100 |
| Editorial Notes | 20, 51, 103 |
| Reviews of Books | 20, 55, 80, 106 |
| Meetings of Societies | 22, 58, 82, 109 |
| Local Medical Notes | 28, 61, 85, 111 |
| Editor's Report | 51 |
| Obituaries | 55, 81, 104 |

### 1949 Index to Vol. LXVI

Ackland, W. R.—Obituary, 81

Arteriography of the Lower Limbs.—H. D. Moore, 37

Baby, Newborn, some Diseases of the.—A. A. Moncrieff, 77

*ac v.* "Early".

Books, Rules for Borrowing by Post, 29

Boycott, J. A.—The Prophylactic Uses of Chemotherapy, 95

Brailsford-Morquio Dwarfism *v.* Unusual Children II

Carey Coombs Memorial Lecture, The Second. The Radiology of Rheumatic Heart Disease.—Sir John Parkinson, 46

Chemotherapy, The Prophylactic Uses of.—J. A. Boycott, 95

Children, Unusual.—A. V. Neale, Beryl D. Corner and J. Lowe, 89

Cholecystitis due to Liver Fluke.—G. P. O'Donnell, 74

Corner, Beryl D.—The Quadruplets: II Management, 14

Corner, Beryl D.—Unusual Children: Cretinism, Mongolism, 89

Cretinism *v.* Unusual Children IV

Domiciliary Consultation.—Editorial Note, 103

Dramatic in Gynæcology and Obstetrics, The.—V. B. Green-Armytage, 69

Drew Smythe, H. J.—Obstetrics and Gynæcology Old and New, 1

Dwarfism *v.* Unusual Children II

Early Life.—A. V. Neale, 31 [cf. Baby].

Editorial Notes, 20, 51, 103
- Veterinary School Buildings, 20
- Medical Library, 20
- Ministerial Dicta, 51
- Domiciliary Consultation, 103
- Unusual Children, 103

Editor's Report, 51

Eyre-Brook, A. L.—The Problem of Cut Flexor Tendons in Fingers, 63

Flexor *v.* Tendons

Gangrene of Extremities *v.* Unusual Children I

Gargoylism *v.* Unusual Children V

Green-Armytage, V. B.—The Dramatic in Gynæcology and Obstetrics, 69

Gynæcology and Obstetrics, The Dramatic in.—V. B. Green-Armytage, 69

Gynaecology, Obstetrics and, Old and New.—H. J. Drew Smythe, 1

Hench, S. Philip.—Psychogenic Rheumatism: Its Differentiation from True Rheumatic Disease, 48

Hospital Economy *v.* Long Fox

Keynes, Geoffrey.—Thymectomy for Myasthenia Gravis, 100

Lees, E. L.—Obituary, 55

Liver Fluke, Cholecystitis due to.—G. P. O'Donnell, 74

Local Medical Notes, 28, 61, 85, 111

Long Fox Memorial Lecture, the Thirty-Seventh, 7

Lowe, J.—Unusual Children: Gargoylism, 89

Medical Library.—Editorial Note, 20

Meetings of Societies, 22, 58, 82, 109

Milnes Walker, R.—The Thirty-seventh Long Fox Memorial Lecture: Some Aspects of Hospital Economy, 7

Milnes Walker, R.—The Surgery of Vascular Disease, 42

Ministerial Dicta.—Editorial Note, 51

Moncrieff, A. A.—Some Diseases of the Newborn Baby, 77

Mongolism *v.* Unusual Children V

Moore, H. D.—Arteriography of the Lower Limbs, 37

Myasthenia Gravis, Thymectomy for.—Geoffrey Keynes, 100

Neale, A. V.—Early Life, 31

Neale, A. V., Corner, Beryl D., and Lowe, J.—Unusual Children, 89

Obituaries :
Edwin Leonard Lees, 55
William Robert Ackland, 81
Geoffrey Commeline Williams, 104

Obstetrics *v.* Gynaecology

O'Donnell, G. P.—Cholecystitis due to Liver Fluke, 74

Parkinson, Sir John.—The Second Carey Coombs Memorial Lecture. The Radiology of Rheumatic Heart Disease, 46

Potter, Mabel F.—The Quadruplets: I Delivery, 14

Progeria *v* Unusual Children III

Prophylactic Uses of Chemotherapy, The. —J. A. Boycott, 95

Psychogenic Rheumatism *v.* Rheumatism

Quadruplets, The.—I. Delivery: H. L. Shepherd and Mabel F. Potter. II. Management: Beryl D. Corner. III. Notes: E. J. Watson-Williams, 14

Radiology of Rheumatic Heart Disease, The—The Second Carey Coombs Memorial Lecture.—Sir John Parkinson, 46

Reviews of Books, 20, 55, 80, 106

Rheumatic Heart Disease *v.* Radiology

Rheumatism, Psychogenic: its Differentiation from True Rheumatic Disease. —Philip S. Hench, 48

Shepherd, H. L. and Mabel F. Potter.— The Quadurplets: I Delivery, 14

Societies, Meetings of:
Bristol M.-Chir. Soc., 22, 82, 108
Clinical Soc. of Bath, 58, 109
Cardiff Med. Soc., 59
Cornwall Clin. Soc., 109
Devon and Exeter M.-Chir. Soc., 26, 60, 83, 109

Societies, Meetings of:—*continued*
Gloucester Med. Soc., 59
Plymouth Med. Soc., 26, 60, 83, 110
Royal Soc. Med. Section Paediatrics, 108
Torquay Med. Soc., 83
West Country Physicians' Club, 83
W. Somerset Med. Club, 60
B.M.A.:
Bath, etc., Branch, 26, 84, 110
S. Wales Branch, 61
Wilts Branch, 84
Barnstaple Div., 84
Bristol Div., 27, 84
Salisbury Div., 85
W. Somerset Div., 85
Trowbridge Div., 85
Anaesthetists, Society of, 83
S.W. Laryngological Assn., 27, 61
Pathologists, Assn. of: Wessex Br. 84

Some Aspects of Hospital Economy, The Thirty-seventh Long Fox Memorial Lecture.—R. Milnes Walker, 7

Some Diseases of the Newborn Baby.— A. A. Moncrieff, 77

Surgery of Vascular Disease, The.— R. Milnes Walker, 42

Syphilis, Congenital, with gangrene, etc. *v.* Unusual Children I

Tendons, Flexor, the Problem of Cut.— A. L. Eyre-Brook, 63

Thymectomy for Myasthenia Gravis.— Geoffrey Keynes, 100

Unusual Children.—A. V. Neale, Beryl D. Corner and J. Lowe, 89

Unusual Children.—Editorial Note, 103

Vascular Disease, The Surgery of.— R. Milnes Walker, 42

Veterinary School Buildings.—Editorial Note, 20

Watson-Williams, E. J.—The Quadruplets: III Notes, 17

Williams, G. C.—Obituary, 104

**Corrigenda :** p. 7. For "Thirty-sixth" read "Thirty-seventh."
p. 14. First line of Dr. Corner's article: for "July" read "June".
p. 52. Sixth line: for "being" read "bring".
p. 76. Thirteenth line: for "poililocytosis" read "poikilocytosis."

### Local Medical Notes

---

HONOURS

**O.B.E.** Wing-Cdr. H. B. Porteous, M.B., Ch.B.

APPOINTMENTS

Professor R. H. Parry has been appointed adviser to the American Mission to Greece, for the reorganization of hospital and health services in that country.

Dr. K. Cowan, County M.O.H. for Essex.

Dr. B. A. Astley Weston, M.O.H., Bath, has been elected President of the West of England branch of the Society of Medical Officers of Health.

Dr. C. D. Evans, O.B.E., M.B., B.Ch., and Mr. R. P. Warin, M.D., M.R.C.P., Dermatologists, United Bristol Hospitals.

Mr. J. R. A. White, M.B., Ch.B., F.R.C.S., Surgeon, West Cornwall Hospital.

Dr. N. R. W. Simpson, M.B., B.S., Director of Physical Medicine, N. Gloucestershire.

Dr. J. A. Pitt Evans, M.B., B.S., B.Sc., Pathologist, Cheltenham.

Dr. J. Macrae, M.D., Physician-in-Charge, Ham Green Hospital, Bristol.

### EXTRACTS FROM REGULATIONS FOR THE MEDICAL LIBRARY AND FOR THE BORROWING AND RETURN OF BOOKS

**Borrowing by Post.**—Any member of the Bristol Medico-Chirurgical Society or subscriber to the *Journal* (being a Registered Medical Practitioner) *residing in Great Britain more than* 10 *miles from the University building*, shall have the right to borrow books from the Medical Library subject to the following provisions :

1. The member or subscriber wishing to borrow a book shall apply in writing to the Superintendent, The Medical Library, The University, Bristol 8.
2. He shall state clearly, for a book, the author, title and year, and for a periodical, the title, volume and date. (All names in BLOCK CAPITALS.)
3. He shall add an undertaking to return the book(s) in good order and to make good any loss or damage.
4. He shall enclose sufficient money for packing and postage ; one shilling for one book, and a further 6d. for each additional book—a heavy volume to be counted as two.
5. He shall return the books, securely packed, by Registered Post, addressed to the Superintendent (as above) at once if requested, and in any case not more than three weeks from the day of despatch.

### MEDICAL DINNER

A medical reunion dinner has been arranged, to be held on 20th April, 1949 : for members of the Bristol Medico-Chirurgical Society and all former students of the Bristol Medical School. Will those who wish to attend please notify the Secretary of the Society at once ? It will be a great help if those who read this notice will bring it to the attention of any former students they may know.

### BRISTOL HOSPITALS' RUGBY CLUB

The Bristol Medical Rugby Club has been refounded. During the war years it had completely lapsed. For many years previous to 1940 there was a flourishing Club connected with the local Medical School, and the Royal Infirmary and General Hospital ran separate teams, which met annually in a memorable series of matches. The present Club has been named The Bristol Hospitals' Rugby Club, and the officers are : Hon. President, Mr. W. M. Capper ; Hon. Treasurer, Mr. Ashton Miller ; Captain, Mr. M. E. Hincks. Membership is open to anyone connected with the Hospitals of Bristol in any way. The side has been considerably helped by many postgraduates, and the junior staff is well represented in most of the games. The Club has already played several local teams, and fixtures are planned with Hospital sides at Oxford and in London. The team settled down well from the beginning of the season, and the match record to date is : Played, 12 ; won, 5 ; lost, 5 ; drawn, 2.

### DEGREES AND DIPLOMAS

**University of Bristol.—**

M.B., CH.B.—R. J. Carey, Anne B. Cousins, Vera H. M. Dowling, D. N. FitzGerald, Margaret Ford, Anne M. French, B. T. Hale, G. A. Hanks, Kathleen J. Harrison*, Joan A. Higginbottom, Una P. Jones, A. H. Laxton, A. J. Lee, D. B. Peacock, Elizabeth Preston-Thomas, G. T. Salter, P. J. Speller, G. D. Teague, A. S. Wallace, Pamela L. C. Watson, Maro A. Wells.

B.D.S.—D. C. Sara.

D.P.M.—M. A. X. Cocheme, V. G. Crotty, W. A. Heaton-Ward.

L.D.S.—D. Clifford, L. B. Howell, H. T. Ivory, J. P. B. Pengelly.

D.M.R.D.—D. M. P. R. Clarke. (We desire to apologize to the practitioner, whose name was wrongly given in the last issue, for what we hope was only intelligent anticipation.)

[* With Distinction in Public Health.]

**University of Cambridge.** M.D. O. C. Lloyd, P. D. Samman.

**Royal College of Surgeons.** F.F.A. Dr. G. L. Feneley.
F.D.S. Mr. John Wells.

